# The Metabolic Syndrome and Risk of Chronic Kidney Disease: Pathophysiology and Intervention Strategies

**DOI:** 10.1155/2012/652608

**Published:** 2012-02-22

**Authors:** Heather A. LaGuardia, L. Lee Hamm, Jing Chen

**Affiliations:** ^1^Tulane University, New Orleans, LA 70112, USA; ^2^Tulane School of Medicine, Tulane University 1430 Tulane Avenue, SL-45, New Orleans, LA 70112, USA

## Abstract

Metabolic syndrome is characterized by a clustering of cardiovascular risk factors, including abdominal obesity, elevated blood pressure and glucose concentrations, and dyslipidemia. The presence of this clinical entity is becoming more pervasive throughout the globe as the prevalence of obesity increases worldwide. Moreover, there is increased recognition of the complications and mortality related to this syndrome. This paper looks to examine the link between metabolic syndrome and the development of chronic kidney disease.

## 1. Introduction

Metabolic syndrome refers to a cluster of metabolic abnormalities (abdominal obesity, hyperglycemia, dyslipidemia, and hypertension) related to a state of insulin resistance, often associated with an overweight or obese state. This clinical entity has been known to increase the risk of cardiovascular disease (CVD), type 2 diabetes, chronic kidney disease (CKD), and total mortality. 

Metabolic syndrome is highly prevalent worldwide, with a prevalence ranging from 10 to 40% in different populations [1–3]. Recently emerging data have suggested that metabolic syndrome is an important risk factor for CKD. CKD is a major risk factor for CVD and premature death [4–7]. Better understanding of the underlying pathophysiology of metabolic syndrome related to CKD will help to identify potential treatment strategies to reduce CKD risk. The purpose of this paper is to explore the potential pathophysiology and treatment strategies related to metabolic syndrome and CKD by integrating available data from the literature.

## 2. Definition of Metabolic Syndrome

In 1923, Kylin [8] first described a constellation of metabolic disturbances that included hypertension, hyperglycemia, and hyperuricemia. Later scientists noted that, when these syndromes clustered together, they could have disastrous health consequences and referred to the clustering as syndrome X, insulin resistance syndrome, the deadly quartet, and obesity dyslipidemia syndrome [9–13]. Between 1998 and 2005, three definitions of metabolic syndrome had been developed and widely used (Table 1). The three definitions stated that the primary components of the syndrome included central obesity, dyslipidemia, elevated blood pressure, and increased glucose. Furthermore, previous studies have reported that all three definitions will identify persons at increased risk for diabetes, cardiovascular disease, and all-cause mortality [3, 14–16]. However, it is noteworthy that the NCEP ATP III definition was a more powerful predictor of CVD and diabetes than the IDF definition [17–20], while the IDF definition identified more patients than the NCEP ATP III definition [19, 21] according to recent studies. Therefore, NCEP ATP III definition may have had more clinical impact. In 2009, a global definition was developed by multiple organizations including the International Diabetes Federation (IDF), National Heart, Lung, and Blood Institute (NHLBI), the World Heart Federation, the International Atherosclerosis Society, and the American Heart Association (AHA) in an effort to harmonize clinical diagnosis of metabolic syndrome. Their definition, summarized in Table 2, is the same as that by NCEP ATP III with an exception that the criteria for elevated waist circumference are based on population- and country- specific definitions [22].

## 3. Metabolic Syndrome and Risk of Chronic Kidney Disease

In the Last ten years, new research has examined the link between kidney disease and metabolic syndrome. In 2004, Chen et al. [23] showed that metabolic syndrome was an independent risk factor of CKD. They examined the association of metabolic syndrome and risk of CKD in over 6000 subjects who participated in the Third National Health and Nutrition Examination Survey (NHANES III) and documented that metabolic syndrome was independently associated with risk of CKD. In 2005, Kurella et al. [24] went further to include all metabolic syndrome traits in relation to risk for CKD. Using data from the ARIC study, a prospective longitudinal study of CV disease risk factors in 10,096 middle-aged nondiabetic adults, they found that over a nine-year time span metabolic syndrome increased the risk of developing chronic kidney disease by approximately 50%. The multivariable-adjusted odds ratio of developing CKD in those with metabolic syndrome was 1.43 (95% CI 1.18–1.73). They also looked at the individual traits associated with the syndrome and found that compared with an adult who has no metabolic syndrome traits, risk for CKD in someone with all five of the traits is two and a half times higher. In 2006, Ninomiya et al. examined the relationship between metabolic syndrome and CKD [25]. They performed a slope analysis of the association between the glomerular filtration rate (GFR) slope and metabolic syndrome by using a multiple regression model. GFR decreased significantly faster in patients with 4 or more metabolic syndrome components compared with those who had 1 or no components. In 2007, Tozawa et al. [26] conducted a prospective study to examine metabolic syndrome as a risk factor for CKD in an Asian population. They examined CKD in 6,371 subjects without CKD or diabetes mellitus at baseline from 1997 through 2002 in Okinawa, Japan. During the 5-year followup, 369 (5.7%) participants developed CKD. After adjusting for age, sex, current cigarette smoking, and alcohol drinking habits at baseline, the relative risk of developing CKD was 1.86 (95% confidence interval: 1.43–2.41, *P* < 0.0001) in subjects with metabolic syndrome.

## 4. Pathophysiology of Metabolic Syndrome which Predisposes to CKD

### 4.1. Insulin Resistance

Insulin resistance has been considered an important pathophysiological factor for metabolic syndrome [27]. Insulin resistance has traditionally been defined by defective insulin action resulting in fasting hyperinsulinemia. Yet, even before fasting hyperinsulinemia develops, postprandial hyperinsulinemia exists. The resultant hyperinsulinemia stimulates glucose uptake by muscle and suppresses endogenous glucose production in the liver. In insulin-resistant conditions, the ability of insulin to augment glucose uptake and inhibit hepatic glucose production is impaired. This creates a state of hyperglycemia that stimulates beta cells to secrete large amounts of insulin postprandially. High insulin concentration may overstimulate the cells of the arterial wall in the skeletal muscle.

Binding of insulin to the insulin receptor normally leads to activation of its tyrosine kinase activity and autophosphorylation of specific tyrosine residues of the receptor. The activated insulin receptor phosphorylates tyrosine residues on substrate proteins initiating a signaling cascade. The two major pathways for insulin signaling are the phosphatidylinositol-3 kinase (PI-3K) and the mitogen-activated protein (MAP) kinase pathways [28]. The PI-3K pathway is initiated by tyrosine phosphorylation of a member of the insulin receptor substrate family, which is associated with the p85 regulatory subunit leading to activation of the enzyme. PI-3K causes phosphatidylinositol 3,4,5-phosphate (PIP3) to be produced. This results in activation of Akt and downstream effector molecules that mediate metabolic response to insulin. This includes translocation of the glucose transporter type 4 (GLUT4) into the membrane. The MAP kinase pathway begins with phosphorylation of insulin receptor substrate, which binds Grb2 and activates Ras. Ras then binds and disinhibits Raf, which activates MEK1 kinase. MEK1 activates extracellular signal-regulated kinases ERK1 and ERK2. The ERKs mediate the mitogenic and proinflammatory responses of insulin signaling. In metabolic syndrome and type 2 diabetes, the pathways leading to activation of PI-3K are blocked, possibly through serine phosphorylation of the insulin receptor, leaving the MAP kinase pathway open. The activation of ERK MAPK pathway stimulates smooth muscle cell growth and proliferation, which maintains normal sensitivity to insulin even in insulin-resistant conditions. The overall effect may be to enhance atherogenesis.

Another key feature of metabolic syndrome is that free fatty acid production and release from adipocytes are not suppressed normally with the usual levels of insulin. Adipocyte resistance to the antilipolytic effect of insulin and the consequent elevated plasma free fatty acid levels may play an important role in the development of insulin resistance in muscle and other target tissues. Furthermore, excess fatty acid blocks the PI-3K signaling pathway. Impairment in the PI-3K pathway could contribute to vascular endothelial dysfunction due to decreased nitric oxide [27–29].

Kubo et al. examined the effect of hyperinsulinemia on renal function in a general Japanese population [30]. The study examined 2446 residents of a town in Japan age 40–79 without renal failure and had them undergo a series of physical and laboratory analyses including glucose tolerance test. The results were interpreted through correlation analysis and showed serum insulin, blood pressure, total cholesterol, low-density lipoprotein cholesterol, triglycerides, and body mass index were all negatively correlated with the reciprocal of serum creatinine level. In multiple regression analysis, the correlation between the sum of insulin levels and the reciprocal of serum creatinine remained significant even after controlling for age, sex, body mass index, blood pressure, total cholesterol, high-density lipoprotein cholesterol, low-density lipoprotein cholesterol, triglycerides, alcohol intake, and smoking habits. This study suggested that hyperinsulinemia was a significant relevant factor of renal function in the general population. Renal dysfunction from insulin resistance and hyperglycemia is thought to be associated with the activation of the renin-angiotensin system (RAS) leading to elevated angiotensin II and aldosterone levels. The elevation affects the insulin/insulin-like growth factor-1 signaling pathways, causing oxidative stress leading to endothelial disruption, and even the development of CVD [31]. Insulin resistance and hyperinsulinemia are associated with decreased endothelial production of nitric oxide and increased oxidative stress which have been also implicated in the progression of diabetic nephropathy [32].

### 4.2. Obesity and Waist Circumference

Visceral adipose tissue is the abdominal fat of the mesentery and omentum. When free fatty acids are released from the visceral fat, they drain into the portal circulation. It has been postulated that increases in this type of fat are directly associated with increases in risk for the sequel of metabolic syndrome [33]. In addition, increases in abdominal subcutaneous fat would release lipolysis products into the systemic circulation and avoid direct effects on hepatic metabolism (i.e., glucose production, lipid synthesis, and secretion of prothrombotic proteins such as fibrinogen and plasminogen activator inhibitor 1) [34]. Furthermore, human adipocytes produce an as yet unidentified mineralocorticoid-releasing factor that stimulates adrenal aldosterone production by means of paracrine or endocrine mechanisms [35, 36]. Elevated levels of aldosterone promote insulin resistance and hypertension and therefore the development of the metabolic syndrome [37].

When biopsies of obese patients are examined, focal and segmental glomerulosclerosis and glomerulomegaly are the most common morphological renal lesions [38]. Early changes noted upon review of biopsies seen in nondiabetic patients with only mild metabolic abnormalities and mild hypertension include increased glomerular cell proliferation, increased mesangial matrix, thicker basement membrane, and increased expression of glomerular transforming growth factor-beta [39]. The mechanisms of obesity-induced renal injury likely result from a combination of hemodynamic and metabolic abnormalities. Many factors contribute to the increase in both glomerular filtration rate (GFR) and rise in renal plasma flow (RPF) observed in obese patients. Insulin resistance likely causes an increase in the efferent arteriolar pressure due to decrease of noradrenaline-induced efferent arteriolar constriction by insulin. Therefore, the transcapillary pressure gradient increases resulting in hyperfiltration [40]. Insulin also stimulates the synthesis of IGF-1 and IGF-2, both promoting glomerular hypertrophy [38]. In 2011, Mathew et al. proposed that circulating cytokines (leptin, adiponectin) and inflammatory markers produced by adipose tissue are directly affecting cells in the renal glomeruli [28]. Moreover, elevated aldosterone in obesity promotes fibrosis and target-organ dysfunction by stimulating plasminogen activator inhibitor, transforming growth factor *β*1, and reactive oxygen species (ROS) [41–43]. Aldosterone also promotes loss of glomerular podocytes and a consequent decrease in the slit-pore membrane integrity, with consequent proteinuria [44–47]. In addition, aldosterone increases renal tubular and interstitial oxidative stress and inflammation, processes that promote salt-induced tubuloglomerular injury, by means of rapid nongenomic effects [28].

### 4.3. Dyslipidemia

This condition is characterized by an increase in elevated triglycerides (and increased VLDL particle number), increased small LDL particles, and low HDL cholesterol. Increased numbers of VLDL and LDL particles lead to an increased level of total apo-B usually observed with atherogenic dyslipidemia. Additionally, small triglyceride-rich lipoproteins have also been found to be atherogenic [48]. The LDL particles associated with the metabolic syndrome and atherogenic dyslipidemia tend to be small and dense. Smaller LDLs have been postulated to penetrate more easily into the arterial wall as well as be more prone to atherogenic modification [49]. Low HDL is a risk predictor for the atherogenic process.

Dyslipidemia seen in metabolic syndrome is postulated to cause CKD by inflammation and increased oxidative stress, which would cause endothelial damage and atherosclerosis diseases [50–52]. Manttari et al. used meta-analysis to postulate that elevated triglycerides and low HDL cholesterol in the plasma are independent risk factors for the development of chronic kidney disease [53]. Additionally, Muntner et al. [54] noted in the ARIC study that high triglycerides and low HDL cholesterol in plasma significantly increased the probability of developing renal dysfunction. It has even been examined that use of statins may slow the progression of chronic kidney disease [55].

### 4.4. Elevated Blood Pressure

Obese persons have a higher prevalence of elevated blood pressure than lean persons. Moreover, a higher blood pressure is a strong risk factor for cardiovascular disease [56]. Well-known complications of hypertension are CHD, stroke, left ventricular hypertrophy, heart failure, and chronic renal failure. The relation between insulin resistance and hypertension is well established [57]. Insulin is a vasodilator when given intravenously to people who are not obese [58]. In the setting of insulin resistance, the vasodilatory effect of insulin can be lost [59], but the renal effect on sodium reabsorption preserved. Metabolic syndrome is also implicated in salt-sensitive hypertension. The enhanced insulin resistance in CKD may increase sodium reabsorption by hyperinsulinemia. This results in sodium retention and salt-sensitive hypertension. In addition, Fujita [60] noted that, in obese rats, adipocyte-derived aldosterone releasing factors lead to hyperaldosteronism. Hyperaldosteronism results in salt-sensitive hypertension as well as proteinuria in the obese hypertensive rats. Salt loading exacerbated the proteinuria and also resulted in cardiac diastolic dysfunction. Fujita proposed that salt and aldosterone worked in synergy with the cardiovascular system through overproduction of oxidative stress. ROS, induced by adipokines such as tumor necrosis factor-alpha, nonesterified fatty acids, angiotensinogen activated the mineralocorticoid receptor, in an aldosterone-independent fashion. The hypothesis proposed aldosterone and mineralocorticoid receptor activation may play an important role in the development of salt-sensitive hypertension, as well as the cardiovascular and renal injury seen in metabolic syndrome. Fatty acids themselves can mediate relative vasoconstriction [61].

## 5. Interventions

### 5.1. Diet

Some experts debate the clinical utility of aggregating individual risk factors into a specific diagnosis of metabolic syndrome when medically each risk factor is addressed separately. At this time, there is no single metabolic syndrome diet recommended. The main strategy has been to reverse contributory factors such as an atherogenic diet, obesity, and a sedentary lifestyle [62]. Weight management and physical activity are recommended as first-line therapy in order to delay the progression of symptoms [63]. Epidemiological evidence suggests a lower prevalence of metabolic syndrome with dietary patterns that are rich in fruits, vegetables, whole grains, dairy products, and unsaturated fats. Research from the dietary approaches to stop hypertension (DASH) intervention studies demonstrates beneficial effects of an eating plan rich in low-fat dairy foods, fruits, and vegetables on blood pressure and lipids. A reduced calorie DASH diet compared to a control and weight loss diet reduced most of the metabolic syndrome risks in both men and women and improved some components beyond that seen in a weight loss diet [64]. The DASH diet which is rich in calcium, magnesium, and potassium may also lower the risk of stroke and hypertension. Fiber and other phytonutrients in fruit and vegetables may be protective by lowering cholesterol or markers of inflammation. Some studies [65, 66] suggest an inverse association between dairy consumption and risk for metabolic syndrome. In young overweight adults, the incidence of metabolic syndrome were lower by more than two-thirds among individuals in the highest category of dairy intake (>5 servings per day) compared to lowest category (<1.5 servings a day) [67]. In addition, a dietary pattern that had higher intake of low-fat dairy has been associated with a lower risk of type 2 diabetes in middle aged or older women and with a 9% lower risk for type 2 diabetes in men [65]. Giugliano et al. explored possible mechanisms underlying a dietary intervention and randomly assigned 180 patients (99 men and 81 women) with the metabolic syndrome to either a Mediterranean-style diet (an increase in daily consumption of whole grains, vegetables, fruit, nuts, and olive oil) or a cardiac diet with a decreased fat intake composed of less than thirty percent of total calories. Only forty patients in the intervention group still had metabolic syndrome after two years compared with seventy-eight patients who consumed the control diet [68].

Given the increased blood pressure reactivity to dietary salt in patients with metabolic syndrome, a reduction in dietary salt may have a beneficial effect on lowering systolic blood pressure as suggested by Hoffman's study (8.2 g/day to 2.3 g/day of salt) [69]. Recently, Chen et al. reported that metabolic syndrome may enhance blood pressure response to sodium intake in nondiabetics [70]. However, if low-sodium diet could lead reduction of metabolic syndrome-related morbidity and mortality remains to be investigated in clinical trials.

### 5.2. Oral Hypoglycemic Agents

In the Diabetes Prevention Program trial, metformin reduced the risk of diabetes and the metabolic syndrome in individuals with impaired fasting glucose and impaired glucose tolerance [71]. The study examined 3234 nondiabetic persons with elevated fasting and postload plasma glucose concentrations. Then assigned participants to metformin (850 mg twice daily) or a lifestyle-modification program and followed them for 2.8 years. The lifestyle intervention reduced the incidence by 58% (95% CI, 48%–66%) and metformin by 31% (95% C, 17%–43%), as compared with placebo; the lifestyle intervention was significantly more effective than metformin. In a 10-year followup of this study, it was found that prevention or delay of diabetes with lifestyle intervention or metformin could persist for at least 10 years [72]. In patients with the metabolic syndrome but normal glucose tolerance, metformin has been shown to improve endothelial function [73]. Unfortunately, metformin is contraindicated in patients with chronic kidney disease with reduced GFR. This is due to the fact that renal clearance of metformin and lactate is reduced, leading to increased levels of both and possibly causing lactic acidosis due to a buildup of lactic acid [74].

Thiazolidinediones have also been associated with protection effects. In the DREAM study, rosiglitazone reduced the three-year incidence of type 2 diabetes by 60 percent in patients with impaired glucose tolerance or impaired fasting glucose who were taking the medication at the time of testing [75]. The management of insulin resistance with thiazolidinediones (TZDs) has resulted in greater attention to activators of the peroxisome proliferator-activated receptors (PPARs). TZDs exert much of their effect on insulin resistance via activation of PPAR-gamma. TZDs not only improve glucose control but favorably affect both free fatty acid metabolism and insulin action. Szapary et al. examined the effect of pioglitazone in patients with the metabolic syndrome and demonstrated a significant increase in HDL-C and favorable effects on lipid subfractions without an effect on triglycerides or low-density lipoprotein cholesterol (LDL-C) concentrations [76]. Tan et al. [77] noted that pioglitazone therapy resulted in greater improvements in the atherogenic index of plasma and lowered triglyceride levels effectively while achieving greater increases in HDL-C when compared with rosiglitazone.

### 5.3. Lipid-Lowering Agents

The efficacy of statins in making marked reductions in LDL cholesterol, modest reductions in TG, and small increases in HDL cholesterol is well documented. ATP III recommended a goal serum LDL cholesterol of less than 100 mg/dL (2.6 mmol/L) for secondary prevention in patients with type 2 diabetes [78, 79]. For this reason the use in patient with metabolic syndrome has been examined. Patients in the 4S trial who met the lipid criteria for the metabolic syndrome were treated with simvastatin, 20 or 40 mg/d. It was noted that they had a 37.5% versus a 36.0% decrease, respectively, in LDL cholesterol, a 24.1% versus 6.7% decrease in TG, and a 10.3% versus a 0.6% increase in HDL cholesterol [80]. Rosuvastatin, 10 mg/d administered to patients with the metabolic syndrome, reduced LDL cholesterol by 47%, apolipoprotein B by 37%, and TG by 23%, while increasing HDL cholesterol by 10% [81]. Multiple statins have been shown to reduce cardiovascular events in patients with and without CVD, suggesting that this is a class effect of these drugs. Some of the CVD risk reduction produced by statins may be attributable to nonlipid pleiotrophic effects of these drugs [82]. Treatment of patients with known coronary disease and the metabolic syndrome with atorvastatin 80 mg, compared to atorvastatin 10 mg, decreased the rate of major cardiovascular events at five years (9.5 versus 13 percent, HR 0.71, 95% CI 0.61–0.84) [79].

In 2009, Robinson et al. [83] evaluated the lipid-lowering efficacy of ezetimibe/simvastatin 10/20 mg versus atorvastatin 10 or 20 mg, and ezetimibe/simvastatin 10/40 mg versus atorvastatin 40 mg in 1,128 patients with hypercholesterolemia and the metabolic syndrome. They noted that greater improvements in the levels of LDL cholesterol, non-high-density lipoprotein cholesterol, apolipoprotein B, and lipid/lipoprotein ratios resulted with ezetimibe/simvastatin compared with atorvastatin at all specified dose comparisons (*P* < 0.001).

### 5.4. Antihypertensive Therapy

Patients with hypertension and the metabolic syndrome have high risk of suffering from future cardiovascular and kidney disease. At present there are no large-scale, randomized trials to establish the antihypertensive drug of choice for patients with metabolic syndrome. Most investigators have considered angiotensin-converting enzyme inhibitors (ACEI) as superior to beta-blockers and thiazide diuretics [84]. The adverse potential metabolic side effects of thiazides and beta-blockers on increase of blood lipids and glucose have led to favoring of ACEI and calcium channel blockers (CCB) [85]. Beta-blockers also promote weight gain, and both thiazides and beta-blockers are associated with an increased incidence of diabetes, compared to CCB and ACEI [86].

Wright et al. [87] conducted subgroup analysis of the ALLHAT study reporting that findings fail to support the preference for CCBs, alpha-blockers, or ACEIs compared with thiazide-type diuretics in patients with the metabolic syndrome. Wright examined the metabolic and cardiovascular outcomes of the ALLHAT trial in patients stratified according to race (black versus nonblack) and the presence or absence of the metabolic syndrome. Among all the patients studied, chlorthalidone had the least favorable effects on blood glucose and cholesterol levels than lisinopril, amlodipine. In patients without the metabolic syndrome, both the ACEI and the CCB lowered the incidence of type 2 diabetes significantly, compared to the thiazide [88]. Wright also noted that black patients with the metabolic syndrome had worse outcomes with lisinopril, compared to chlorthalidone, with respect to every outcome measured, likely resulting from a 3–5 mmHg higher systolic pressure in the black patients on lisinopril, compared to those on chlorthalidone. Wright concluded that ACEI should not be the first-line monotherapy for black patients with the metabolic syndrome [87].

### 5.5. Resistance Training

Over the last decade, physicians have been examining the effects of resistance training on metabolic syndrome. Reduced muscle mass as a result of normal aging and decreased physical activity have been postulated behind the high prevalence of this disorder. Improved glycemic control, improved blood lipid profiles, and decreased BP are important for reducing microvascular and macrovascular complications in people with metabolic risk. As with increasing adiposity in aging and loss of muscle mass, the insulin-mediated glucose uptake and TG disposal in the skeletal muscle of elderly persons is reduced and the maintenance of a large muscle mass can contribute to the prevention of type 2 diabetes, which is associated with cardiovascular disease. Resistance training is contributing to the decrease of major risk factors for the metabolic syndrome and should be recommended for the management of type 2 diabetes. Although the number of studies on the effects of resistance training on blood pressure is small, Strasser conducted meta-analysis confirming that resistance training does not increase blood pressure as was once thought and may even have potential benefits on resting systolic blood pressure [89].

### 5.6. Surgery

In the prospective controlled clinical study conducted by Lee et al., metabolic syndrome was prevalent in 52.2% of morbidly obese individual enrolled. Significant weight reduction 1 year after surgery markedly improved all aspects of the metabolic syndrome and resulted in a cure rate of 95.6% [90]. Obesity surgery performed by laparoscopic surgery is recommended for obese patients with the metabolic syndrome that have not responded to conservative measures.

## 6. Conclusion

Compelling data have indicated that metabolic syndrome increases the risk of CKD. Experimental studies have suggested that metabolic syndrome may induce CKD via multiple mechanic pathways. While we are waiting for randomized clinical trial and even new drug development in treating metabolic syndrome to reduce risk of CKD, current key strategies should include prevention and treatment of obesity and insulin resistance. Lifestyle modification particularly including low sodium diet and increasing physical activity would be important approaches. Aldosterone antagonists would also be particular of interest to test in clinical trial in treating metabolic syndrome to reduce CKD risk. At the present, early identification of metabolic syndrome and treatment of individual components of metabolic syndrome may reduce the risk of CKD. However, these approaches need be further tested in large randomized clinical trial to verify their effect on reducing CKD risk.

## Figures and Tables

**Table 1:** 

Parameters	WHO (1998)	NCEP ATP3 (2001)	IDF (2005)
Required	Insulin resistance in the top 25%; glucose		Waist >94 cm (men) or 80 cm (women)

Number of abnormalities	>2	>3	>2

Glucose	>6.1 mmol/L (110 mg/dL); 2-hour glucose >7.8 mmol/L (140 mg/dL)	>5.6 mmol/L (100 mg/dL) or drug treatment for elevated blood glucose	>5.6 mmol/L or diagnosed diabetes

HDL cholesterol	<0.9 mmol/L (35 mg/dL) (men); <1.0 mmol/L (40 mg/dL) (women)	<1.0 mmol/L men (40 mg/dL) (men); <1.3 mmol/L (50 mg/dL) (women) or drug treatment for low HDL-C	<1.0 mmol/L (40 mg/dL) (men); <1.3 mmol/L (50 mg/dL) (women) or drug treatment for low HDL-C

Triglycerides	≥1.7 mmol/L (150 mg/dL)	≥1.7 mmol/L (150 mg/dL) or drug treatment for elevated triglycerides	≥1.7 mmol/L (150 mg/dL) or drug treatment for elevated triglycerides

Obesity	Waist/hip ratio >0.9 (men) or >0.85 (women) or BMI ≥30 kg/m^2^	Waist ≥102 cm (men) or ≥88 cm (women)	Waist ≥94 cm (men) or ≥80 cm (women)

Hypertension	≥140/90 mmHg	≥130/85 mmHg or drug treatment for HTN	≥130/85 mmHg or drug treatment for HTN

**Table 2:** 

Measure	Categorical cut points
Elevated waist circumference	Population- and country-specific definitions
Elevated triglycerides or drug treatment for elevated triglycerides	≥150 mg/dL
Reduced HDL-C or drug treatment for reduced HDL-C	<40 mg/dL in men, <50 mg/dL in women
Elevated blood pressure or antihypertensive drug treatment	Systolic ≥130 mmHg and/or diastolic ≥85 mmHg
Elevated fasting glucose or drug treatment of elevated glucose	≥100 mg/dL

Adapted from [22].
